# Comparative safety and efficacy of tislelizumab-based regimens versus chemotherapy in lung cancer: a systematic review and meta-analysis

**DOI:** 10.3389/fonc.2025.1628742

**Published:** 2025-10-07

**Authors:** Aman Ullah, Faseeh Haider, Faizan Ahmed, Muhammad Arham, Allah Dad, Haseeb Tareen, Fahad Saleem, Kinza Bakht, Atif Nawaz Malik, Zaima Afzaal, Fatima Binte Athar, Kainat Aman, Hira Zahid, Muhammad Asjid, Saboor Ejaz, Zaheer Qureshi, Moazzam Shahzad

**Affiliations:** ^1^ Saint Louis University, St. Louis, MO, United States; ^2^ Allama Iqbal Medical College, Lahore, Pakistan; ^3^ Department of Internal Medicine, Jersey Shore University Medical Center, Neptune, NJ, United States; ^4^ Sheikh Zayed Medical College/Hospital, Rahim Yar Khan, Pakistan; ^5^ Henry Ford Health, Jackson, MI, United States; ^6^ Punjab Medical College, Faisalabad, Pakistan; ^7^ Ameer-ud-Din Medical College, Lahore, Pakistan; ^8^ Karachi Medical and Dental College, Karachi, Pakistan; ^9^ Batterjee Medical College, Jeddah, Saudi Arabia; ^10^ Department of Pathology, Albany Medical Center, Albany, NY, United States; ^11^ Maimonides Medical Center, New York, NY, United States; ^12^ Frank H Netter MD School of Medicine at Quinnipac University, North Haven, CT, United States; ^13^ Moffit Cancer Center, Tampa, FL, United States

**Keywords:** tislelizumab, chemotherapy, lung cancer, efficacy, safety

## Abstract

**Background:**

Lung cancer is the leading cause of cancer-related mortality worldwide, with its burden expected to rise significantly by 2025. Despite therapeutic advances, survival rates remain low, and comorbidities further complicate management. Economic projections indicate lung cancer will account for the highest share of cancer-related costs through 2050.

**Objectives:**

To evaluate the safety and efficacy of Tislelizumab with or without chemotherapy versus chemotherapy alone in lung cancer by synthesizing available evidence through meta-analysis.

**Methodology:**

This meta-analysis followed PRISMA guidelines and was registered in PROSPERO. A systematic search of PubMed, Embase, Scopus, Cochrane Library, ScienceDirect, and ClinicalTrials.gov was conducted for RCTs comparing Tislelizumab-based regimens to chemotherapy in lung cancer (up to February 2025). Key outcomes included PFS, OS, ORR, DCR, and AEs. Bias was assessed using the Cochrane tool, and data were analyzed with random-effects models, incorporating subgroup and sensitivity analyses. Publication bias was assessed via funnel plots and Egger’s test.

**Results:**

A total of six studies involving 2,148 patients were included in the meta-analysis. Tislelizumab-based regimens showed significant improvements in PFS (HR = 0.62, p < 0.0001) and OS (HR = 0.69, p < 0.0001) compared to chemotherapy alone. The ORR (RR = 1.49, p= 0.0001) and DCR (RR = 1.49, p= 0.0010) were significantly higher in the Tislelizumab group. The Tislelizumab group significantly reduced all-cause mortality (RR = 0.89, p = 0.0003). No significant differences were found in AEs (RR = 1.00, p= 0.75), except for ALT and AST elevations (RR = 1.36; 95% CI, 1.13–1.64) and (RR = 1.77; 95% CI, 1.17–2.67), respectively.

**Conclusions:**

Tislelizumab-based regimens offer significant benefits over chemotherapy in lung cancer, with improved PFS, OS, and ORR. It significantly reduced all-cause mortality; however, the observed increase in ALT and AST underscores the need for vigilant liver function monitoring.

**Systematic review registration:**

https://www.crd.york.ac.uk/PROSPERO/view/CRD42025641055, Identifier CRD42025641055.

## 1 Introduction

Lung cancer is the second most commonly diagnosed cancer, accounting for approximately two million deaths globally (1). According to estimates, this mortality number will increase by 1.7 times by 2025. The American Lung Association reported that Lung cancer caused 1 out of 5 cancer-related deaths in 2021 (2). In addition to its high mortality, lung cancer often coexists with other chronic conditions, such as chronic obstructive pulmonary disease (3), pneumonitis (4), cardiovascular diseases (5), and paraneoplastic syndromes (6) etc, that can complicate management and increase the overall disease burden. Moreover, Chen et al. (7) reported that Lung cancer is projected to contribute the largest share to the economic burden of cancer between 2020 and 2050 and is being estimated to be responsible for nearly 15% of the total cancer-related costs. The above stated issues underscore the need for more novel and effective interventions to deal with this ailment.

Treatment for lung cancer has seen significant improvements over time. In the beginning, traditional methods such as surgery and radiation were primarily implied, followed by chemotherapies, specifically Platinum-based therapies (8). Recently, we have seen the development of more targeted immunotherapies, which include agents like anti-Programmed Cell Death Protein (PD-1) monoclonal antibodies (Nivolumab, Pembrolizumab, and Tislelizumab (9)). Among these advancements, Tislelizumab stands out as a recently developed anti- PD-1 monoclonal antibody. It is designed with the intention of bypassing the resistance mechanisms which are commonly encountered with conventional anti-PD-1 therapy, specifically by minimizing FcyR binding (10). Furthermore, high PD-1 binding affinity, improved tumor microenvironment penetration, and improved outcomes in previously treated non-small cell lung cancer (11) gives it edge over other immunotherapies. The improved end results of Tislelizumab has been further emphasied by the RATIONALE-307 trial, demostrating that when Tislelizumab is administered in combination with chemotherapy, it results in improved Progression-Free Survival (PFS), Objective Response Rate (ORR) and better safety profile as compared to chemotherapy alone. However, the present literature has not extensively looked into the comparative safety and efficacy of Tislelizumab-based regimens against the chemotherapy alone and to fill this knowledge gap, we require more thorough studies on this aspect.

This meta-analysis study is primarily focusing on the systematic evaluation and comparison of the safety and efficacy of Tislelizumab with and without chemotherapy against the chemotherapy alone, across different subtypes of lung cancer. The findings from this study are intended to guide the future clinical management for Lung Cancer therapy.

## 2 Methodology

### 2.1 Protocol

Meta-analysis was made according to the recommendations of the Cochrane Collaboration and the Preferred Reporting Items for Systematic Reviews and Meta-Analyses (PRISMA) statement for systematic reviews and meta-analysis (11, 12). The study protocol was registered with the International Prospective Register of Systematic Reviews (PROSPERO) under the ID CRD42025641055.

### 2.2 Data sources and search strategy

A comprehensive electronic literature search was performed across various databases, including PubMed, Science Direct, Clinicaltrials.gov, Scopus, Cochrane Library, and Embase, from inception until February 2025 to retrieve randomized controlled trials (RCTs), evaluating the effectiveness of Tislelizumab with or without chemotherapy versus chemotherapy in patients with Lung cancer. The search strategy utilized both predefined Medical Subject Headings (MeSH) and free-text keywords in combination with the Boolean operators “AND” and “OR” to create database-specific search strategies. The search approach included the phrases “Lung Neoplasm”, “Lung Cancer”, “Tislelizumab”, “Anti-neoplastic agents”, and “Chemotherapy”. No restrictions on language or time frame were placed. In addition, manual screening of the reference lists of included studies was conducted to identify additional studies. The detailed search strategy for each database is available in (**Supplementary Tables S1-3**).

### 2.3 Eligibility criteria

The inclusion criteria were based on the PICOS format for systematic reviews and meta-analysis, with population (P) being patients with Lung Cancer, intervention (I) being Tislelizumab with or without chemotherapy, control (C) being chemotherapy, and O being several outcomes defined below. Studies that adhered to the following inclusion criteria were considered: (a) randomized controlled trials (RCTs) (b) patients of Lung cancer receiving Tislelizumab with or without chemotherapy in one arm, (c) patients of Lung cancer receiving chemotherapy in the other arm and (d) reported at least one of the outcomes of interest. The outcomes of interest included PFS, ORR, Overall Survival (OS), disease control rate (DCR), duration of response (DOR), and adverse events (AE). The exclusion criteria were: (a) studies categorized as other than RCTs e.g. observational studies, letters to editors/commentaries, case reports, guidelines, literature reviews, systematic reviews, and meta-analyses, (b) studies not comparing Tislelizumab with or without chemotherapy against chemotherapy, (c) studies including patients other than lung cancer and (d) studies missing clinical information relevant to the outcomes being investigated.

### 2.4 Study selection

Before the screening process commenced, all identified studies were removed of duplicates. Two independent reviewers initially screened titles and abstracts for inclusion and excluded articles that did not satisfy the eligibility criteria. Full-text manuscripts of the remaining eligible studies were retrieved and thoroughly examined for inclusion. Any disagreements between the reviewers were resolved by reaching a consensus or discussion with a third independent reviewer.

### 2.5 Data extraction and quality assessment

Data extraction was carefully performed independently by two reviewers using a pre-piloted Microsoft Excel spreadsheet. Any discrepancies were managed by a third reviewer. The data extracted from each eligible study included the author’s name, publication year, country of origin, study design, total sample size in both groups, mean age, male %, type of lung cancer, and the reported outcomes of interest. One of the studies, Wang J et al. (13), had two intervention arms (A: Tislelizumab + Paclitaxel + Carboplatin, B: Tislelizumab + nab-Paclitaxel + Carboplatin) and one comparator arm (C: Paclitaxel + Carboplatin) and hence was subdivided into Wang J et al. (13) A (Arm A versus Arm C) and Wang J et al. (13) B (Arm B versus Arm C) for data synthesis. The Cochrane’s Collaboration tool for assessing Risk of Bias (RoB 1.0) was employed for analyzing bias risk in randomized clinical trials (RCTs) by evaluating following domains: [1] sequence generation (selection bias); [2] allocation concealment (selection bias); [3] blinding of participants and personnel (performance bias); [4] blinding of outcome assessment (detection bias); [5] incomplete outcome data (attrition bias); [6] selective outcome reporting (reporting bias) and [7] other bias. Each domain was categorized as ‘low risk’, ‘unclear risk’, and ‘high risk’ according to the predefined criteria.

Primary outcomes were PFS, OS, DOR, ORR, and DCR. Subgroup analyses were performed by lung cancer subtype, histological classification, and intervention regimen (Tislelizumab monotherapy vs. Tislelizumab combination therapy) to explore potential sources of biological variability and heterogeneity.

### 2.6 Data analysis

All statistical analyses were conducted using R (version 2024.12.1 + 563) with the “meta” and “metafor” packages. Outcomes of Dichotomous variables were expressed as Risk ratios with 95% CI, while time-to-event outcomes were expressed as Hazard Ratios with 95% CI using log-transformed HRs to approximate normal distribution (14), with standard errors. Meta-analyses were performed to pool data and to obtain overall effect sizes. A random effects model was consistently applied for interpretation, in line with current recommendations (15), and the Dersimonian-Laird estimator (τ² or Tau²) was used to estimate inter-study variance. Statistical heterogeneity was evaluated using Higgins I^2^ statistics (16), where values of 0–25% represented low heterogeneity, 25–75% indicated moderate heterogeneity, and values above 75% reflected high heterogeneity. Leave-one-out sensitivity analyses were performed to explore the sources of the potential heterogeneity by excluding one study at a time, allowing us to evaluate the contribution of each study to the overall estimate. Although a minimum of ten studies is typically recommended for publication bias assessment (17), we lowered the threshold to five to enable analysis, given the limited number of studies. Publication bias was evaluated via funnel plot inspection and Egger’s regression test (two-tailed, p < 0.05). When asymmetry was detected, we applied the trim-and-fill method (L-estimator) under a random-effects model using the inverse variance method with REML for τ² estimation.

## 3 Results

### 3.1 Systematic process of study selection

**Figure 1:**
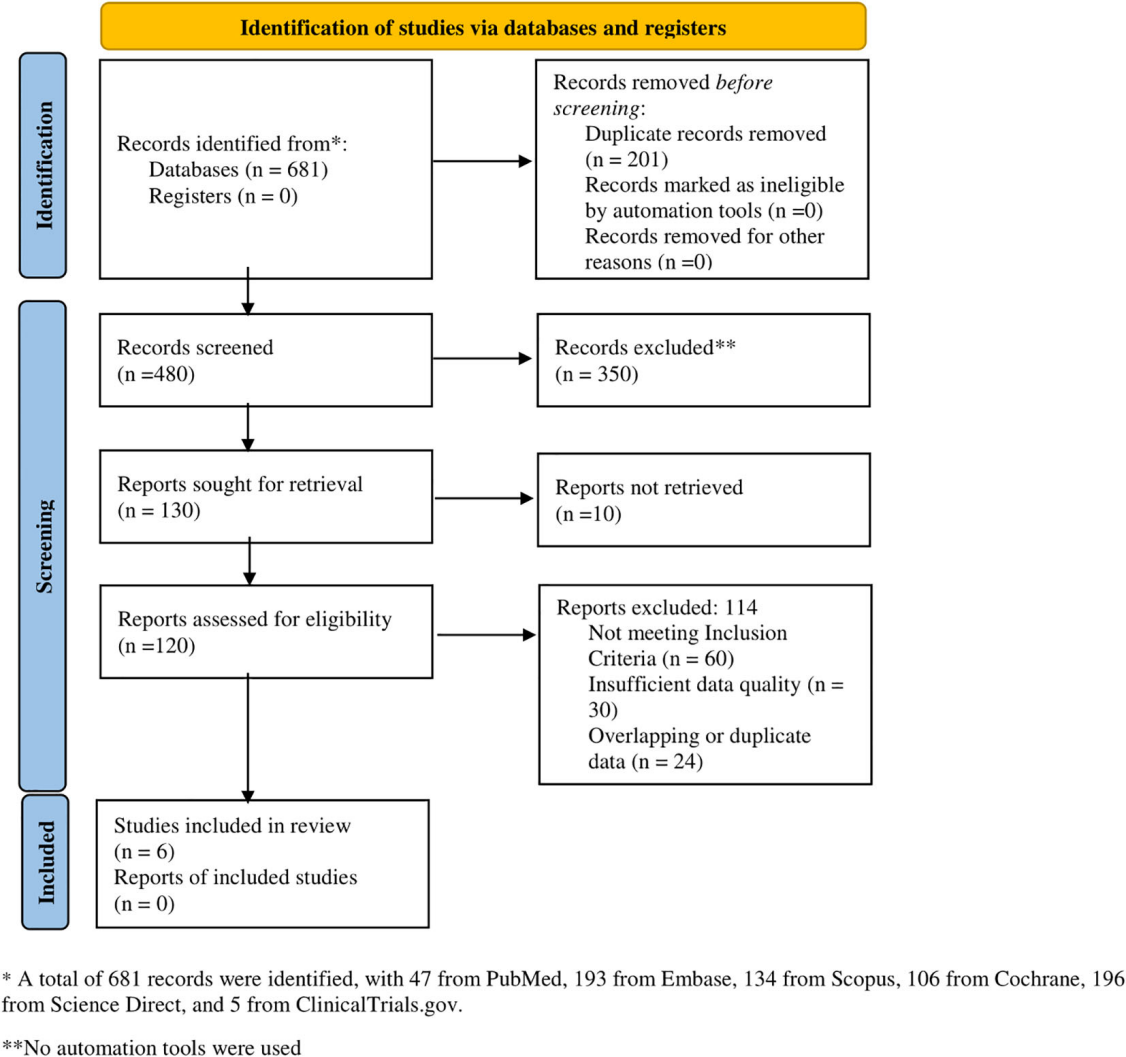
PRISMA flow diagram of the included studies.

Following PRISMA guidelines, a total of 681 records were identified across multiple databases, including PubMed (n = 47), Embase (n = 193), Scopus (n = 134), Cochrane (n = 106), Science Direct (n = 196), and ClinicalTrials.gov (n = 5). After screening 480 records, 350 were excluded. Of the 130 reports sought for retrieval, 10 were unavailable, leaving 120 for eligibility assessment. Among these, 114 were excluded due to unmet inclusion criteria (n = 60), insufficient data quality (n = 30), or duplication (n = 24). Ultimately, six studies were included in the final review (**Figure 1**).

### 3.2 Summary of reviewed articles

A total of six studies were included in this systematic review and meta-analysis, encompassing 2,148 patients, of whom 1,299 (60.5%) were in the treatment group. We compared treatment regimens involving Tislelizumab with or without chemotherapy to chemotherapy alone. **Table 1** summarizes the study and patient characteristics for a detailed understanding of the included trials.

**Table 1:** Baseline table and patient characteristics.

Author Name, Year	Intervention Group	Comparator Group	Disease of Population	N	Gender		Patient Sample (n)		Age in Years	
					Male	Female	Intervention	Comparator	Intervention	Comparator
Zhou C et al., 2022 (10)	Tislelizumab	Docetaxel	NSCLC (mixed)	805	622	183	535	270	61 (28–88)	61 (32–81)
Wang J et al., 2021 (13)	Arm A: Tislelizumab + Paclitaxel + Carboplatin Arm B: Tislelizumab + nab-Paclitaxel + Carboplatin	Paclitaxel + Carboplatin	Squamous NSCLC	360	330	30	239	121	Arm A: 60 (41–74) Arm B: 63 (38–74)	62 (34–74)
Lu S et al., 2021 (18)	Tislelizumab + Platinum-based Chemotherapy	Platinum-based chemotherapy	Non-squamous NSCLC	334	247	87	223	111	60 (27–75)	61 (25–74)
Cheng Y et al., 2024 (19)	Tislelizumab + Platinum and Etoposide	Placebo + Platinum and Etoposide	SCLC	457	372	85	227	230	63 (56–66)	62 (56–67)
Gong Y et al., 2024 (20)	Tislelizumab + Chemoradiotherapy	Chemoradiotherapy	SCLC	126	99	27	42	43	59.9 ± 7.11	61.0 ± 9.54
Li et al., 2022 (21)	Tislelizumab + Pemetrexed	Pemetrexed	Adenocarcinoma	66	29	37	33	33	65.74 ± 2.36	65.58 ± 2.49

NSCLC (mixed): patient cohort comprising both squamous and non-squamous lung cancer histologies.

N, Total Number of Patients; RCT, Randomized controlled trial; SCLC, Small Cell Lung Cancer; NSCLC, Non-small cell lung cancer; Age is either given in median (range) or mean ± SD.

The Risk of Bias (ROB) tool evaluation indicated that the literature included medium to high-quality studies. All studies reported adequate random sequence generation, while four demonstrated appropriate allocation concealment. Two studies properly blinded participants, and no study reported proper blinding of outcome assessors. None of the studies exhibited selective reporting, and all were free from other biases as shown in (**Supplementary Figure S6**).

### 3.3 Primary outcomes

**Figure 2:**
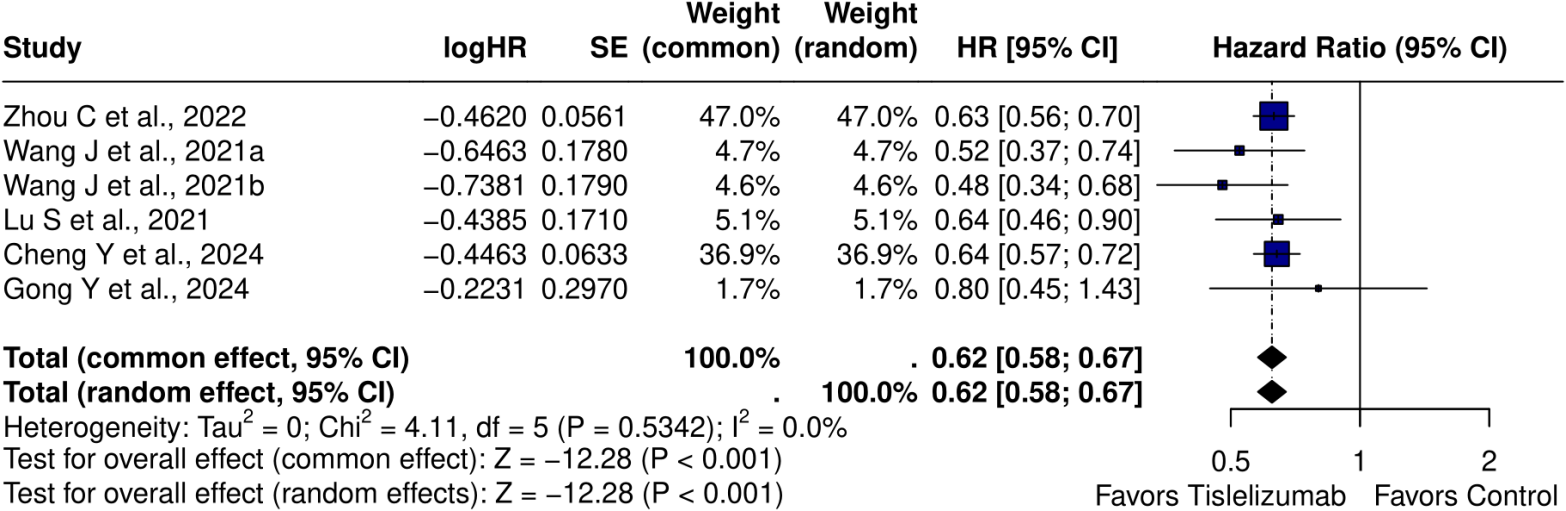
Forest plot of pooled hazard ratios for PFS [HR = 0.62 (95% CI: 0.58-0.67); p < 0.001].

The pooled HR for PFS using a random effects model was 0.62 (95% CI: 0.58–0.67, p < 0.001), indicating a significant benefit of Tislelizumab-based treatment. Heterogeneity was minimal (I² = 0.0%, τ² = 0, p = 0.5342). Egger’s regression test for small-study effects showed no significant publication bias (t = -0.66, df = 4, p = 0.543), with a bias estimate of 0.4935 (SE = 0.7424), suggesting no substantial asymmetry in the funnel plot (**Figure 2**). Subgroup analysis demonstrated that Tislelizumab significantly improved PFS in patients with NSCLC (HR = 0.61; 95% CI, 0.55–0.67; I² = 0.7%) and SCLC (HR = 0.65; 95% CI, 0.57–0.73; I² = 0%) (**Supplementary Figure S1**). Further stratification of NSCLC revealed a pronounced benefit in squamous NSCLC (HR = 0.50; 95% CI, 0.39–0.64; I² = 0%) (**Supplementary Figure S2**). Only one study, Lu et al. (18), reported outcomes for non-squamous NSCLC, showing an HR of 0.64 (95% CI, 0.46–0.90). When analyzed by treatment regimen, Tislelizumab in combination with chemotherapy improved PFS (HR = 0.62; 95% CI, 0.56–0.69; I² = 1%). Excluding Zhou et al. (10), which evaluated Tislelizumab monotherapy, yielded a pooled HR of 0.63 (95% CI, 0.56–0.70) for the monotherapy subgroup. Two studies (21, 22) reported PFS duration in months, comparing the intervention and control groups. The pooled PFS using a double-arm model was 1.30 months (95% CI: 0.26–6.42), with no significant heterogeneity (I² = 0.0%, τ² = 0, p = 0.9816; z = 0.32, p = 0.7476).

**Figure 3:**
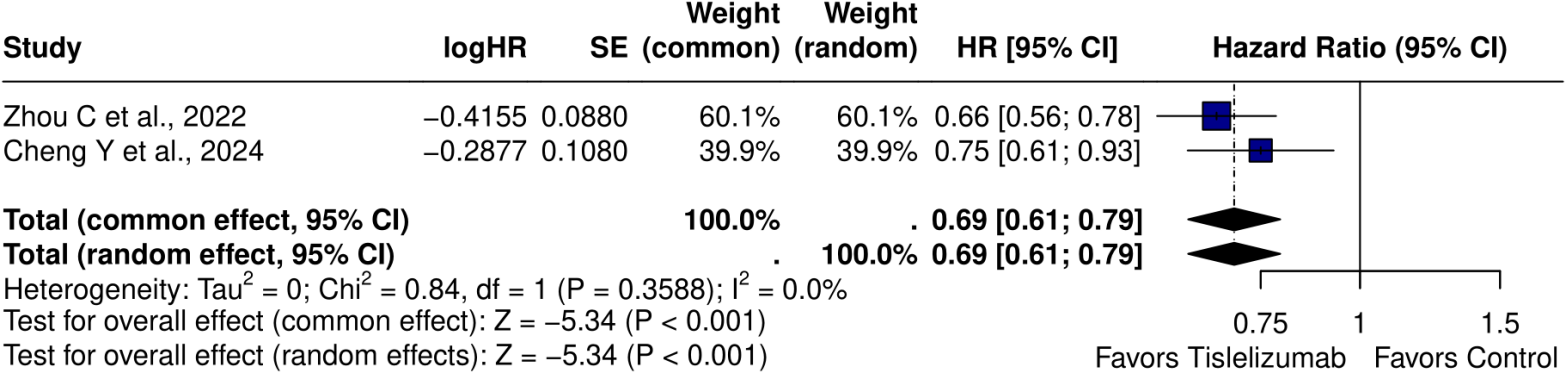
Forest plot of pooled hazard ratios for OS [HR = 0.69 (95% CI: 0.61-0.79); p < 0.001].

Two studies (10, 19) reported HRs for OS and DOR. The pooled HR for OS using a random-effects model was 0.69 (95% CI: 0.61–0.79, p < 0.0001), with no significant heterogeneity observed (I² = 0.0%, τ² = 0, p = 0.3588) (**Figure 3**). In contrast, the pooled HR for DOR 0.66 (95% CI: 0.35–1.25) was comparable to chemotherapy, with substantial heterogeneity (I² = 76.0%, τ² = 0.1660, p = 0.0413). Three studies (10, 18, 19) reported the DOR for the intervention group in months. The pooled DOR using a single-arm model was 7.69 months (95% CI: 4.14–14.44), with significant heterogeneity (I² = 95.2%, τ² = 0.2885, p < 0.0001).

**Figure 4:**
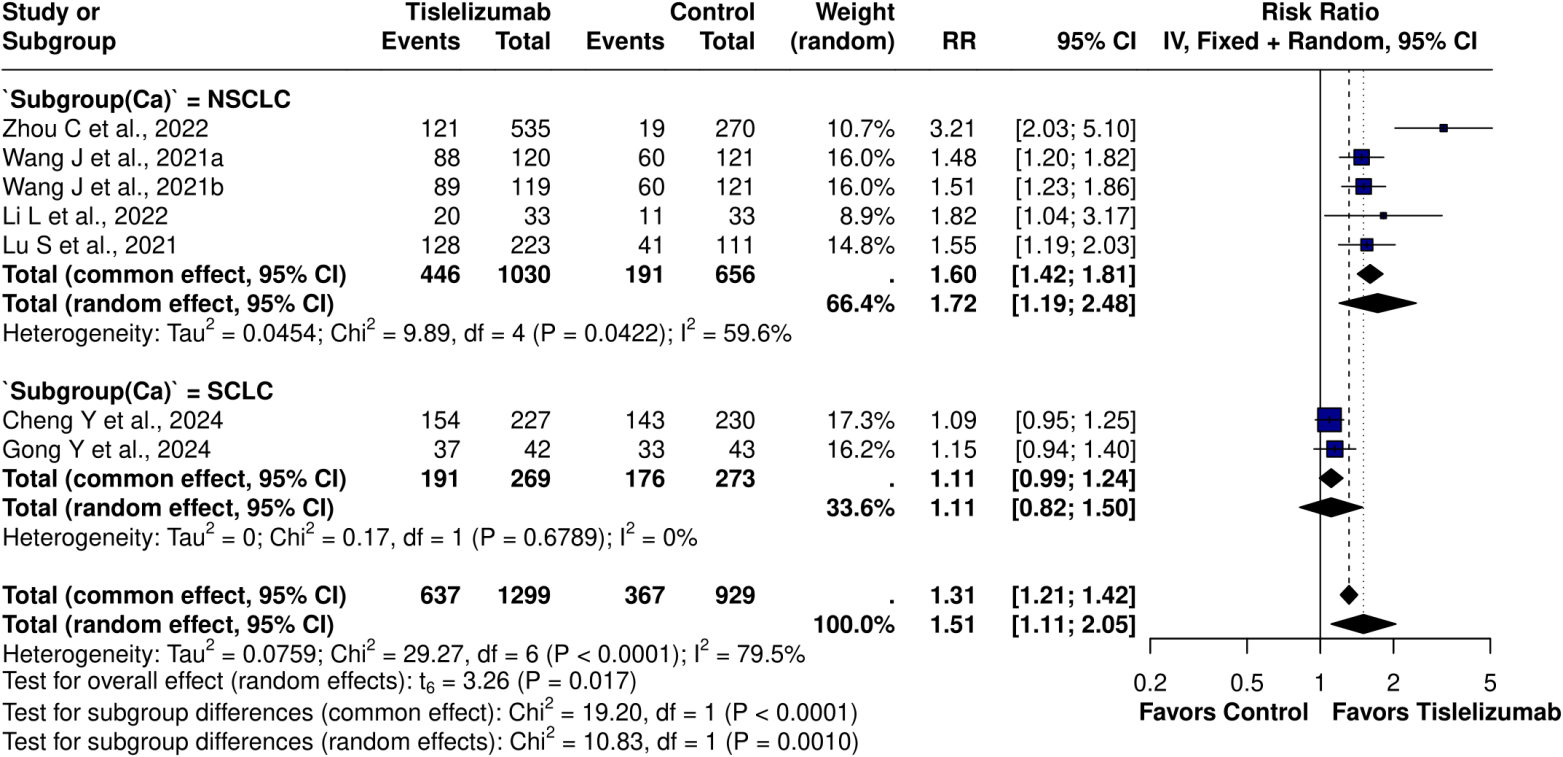
Subgroup forest plot for ORR based on lung cancer subtype [NSCLC: RR = 1.72 (95% CI: 1.19-2.48), I^2^ = 0%; SCLC: RR = 1.11 (95% CI: 0.82 – 1.50), I^2^ = 0%].

ORR was significantly higher in the Tislelizumab group (RR = 1.51; 95% CI, 1.19–1.90; I² = 79.5%), with heterogeneity remaining unchanged in leave-one-out sensitivity analysis. In a pooled analysis of six studies evaluating Tislelizumab combination therapy, the RR was 1.34 (95% CI, 1.11–1.63), although Zhou et al. (10) reported a substantially higher effect for Tislelizumab monotherapy (RR = 3.21; 95% CI, 2.03–5.10) (**Supplementary Figure S3**). The effect was attenuated in SCLC (RR = 1.11; 95% CI, 0.82–1.50; I² = 0%) but remained significant in NSCLC (RR = 1.72; 95% CI, 1.19–2.48; I² = 59.6%) (**Figure 4**) (**Supplementary Figure S4**). Further stratification of NSCLC showed benefit in squamous NSCLC (RR = 1.49; 95% CI, 1.32–1.69; I² = 0%) but no effect in non-squamous NSCLC (RR = 1.60; 95% CI, 0.73–3.50; I² = 0%). DCR was higher with Tislelizumab (RR = 1.49; 95% CI, 1.18–1.90) compared with chemotherapy alone. DCR (t = 3.67; df = 4; p = 0.0213) exhibited statistically significant publication bias; however, interpretation is limited by the small number of studies. Application of the trim-and-fill method imputed three studies, yielding an adjusted RR of 1.06 (95% CI, 0.60–1.88; p = 0.85; I² = 96%), which attenuated the effect.

### 3.4 Safety outcomes

While the overall incidence of adverse events was similar between groups (RR = 1.00, 95% CI: 0.99–1.01, p = 0.7545), all-cause mortality was significantly lower in the Tislelizumab group (RR = 0.89, 95% CI: 0.83–0.95, p = 0.0003), reinforcing its potential survival benefit. Additionally, hepatic toxicities were more frequent in the intervention arm, with a significant increase in ALT (RR = 1.36; 95% CI, 1.13–1.64; I² = 24.0%). This effect was attenuated when analyses were stratified by lung cancer type and histological subtype. The regression test indicated no significant funnel plot asymmetry (p = 0.519). AST was elevated in the intervention arm (RR = 1.77; 95% CI, 1.17–2.67). Omitting Lu et al. (18) reduced heterogeneity to 48.4% (RR = 2.03; 95% CI, 1.45–2.84) (**Supplementary Figure S7**). Subgroup analysis identified the highest risk in squamous NSCLC (RR = 2.91; 95% CI, 2.05–4.15; I² = 0%), which explained the heterogeneity (**Supplementary Figure S5**). Egger’s test indicated funnel plot asymmetry (t = 3.22; df = 4; p = 0.0324). Trim-and-fill imputed three studies, yielding an adjusted RR of 1.20 (95% CI, 0.67–2.14; p = 0.49; I² = 85.3%), attenuating the effect. Other adverse events, including anemia, thrombocytopenia, and neutropenia, did not show statistically significant differences. These findings highlight the therapeutic advantages and potential safety considerations associated with Tislelizumab-based regimens (**Table 2**). Evidence of publication bias was assessed using Egger’s test across multiple clinical outcomes. For adverse events, no significant small-study effects were detected (t = -0.42, df = 4, p = 0.6984), with a bias estimate of -0.6663 (SE = 1.5994). Similarly, anemia (t = -1.01, df = 5, p = 0.3573) and thrombocytopenia (t = 0.93, df = 4, p = 0.4051) demonstrated no significant bias. Similarly, hypoalbuminemia (t = 2.73, df = 4, p = 0.0522) and neutropenia (t = -1.18, df = 5, p = 0.2893) showed no significant bias, with heterogeneity eliminated upon exclusion of Zhou et al. (10).

**Table 2:** Summary of clinical safety outcomes.

Outcomes	N	Intervention Group		Control Group		Risk ratios (95% CI)	Heterogeneity (I²)	P-value
		Events	Total	Events	Total			
Clinical Safety Outcomes								
Adverse Events	2442	1245	1264	880	887	1.00 (95% CI: 0.99, 1.01)	0%	P = 0.7545
Anemia	2508	778	1297	624	920	0.99 (95% CI: 0.88, 1.11)	80.2%	P = 0.8323
Neutropenia	2508	463	1297	445	920	0.72 (95% CI: 0.50, 1.03)	92.1%	P = 0.0731
Febrile Neutropenia	720	5	238	2	234	2.44 (95% CI: 0.47, 12.54)	0%	P> 0.05
Thrombocytopenia	1703	357	762	261	650	1.07 (95% CI: 0.93, 1.23)	17.7%	P = 0.3274
All-cause Mortality	1388	543	804	411	543	0.89 (95% CI: 0.83, 0.95)	0%	P = 0.0003
ALT	2442	377	1264	193	887	1.36 (95% CI: 1.13, 1.64)	24%	P = 0.0006
Hypo-proteinemia	2442	204	1264	113	887	1.24 (95% CI: 0.96, 1.61)	23.1%	P = 0.1004
AST	2442	338	1264	148	887	1.77 (95% CI: 1.17, 2.67)	80.4%	P = 0.0069

N, Number of Patients; ORR, Objective Response Rate; DCR, Disease Control Rate; RR, Risk Ratio; OR, Odds Ratio; CI, Confidence Interval; ALT, Alanine Aminotransferase; AST, Aspartate Aminotransferase.

## 4 Discussion

This meta-analysis demonstrates that tislelizumab-based treatments are more effective than chemotherapy for both small cell and non-small cell lung cancers, including squamous, non-squamous, and adenocarcinoma subtypes. Across a pooled cohort of 2,148 patients, Tislelizumab regimens were associated with longer PFS and OS across different patient subgroups regardless of histological type or whether used alone or in combination, whereas DOR was comparable to chemotherapy. ORR and DCR were also higher with tislelizumab, particularly in NSCLC with squamous histology. The overall side effect profile was comparable between groups, although tislelizumab was linked to a higher likelihood of liver enzyme elevations. Importantly, tislelizumab treatment was associated with lower mortality, underscoring its clinical benefit over chemotherapy.

Historically, treatment guidelines for advanced NSCLC held chemotherapy (e.g., taxanes or platinum-based agents) as the mainstay of first-line therapy, reserving immune checkpoint inhibitors like nivolumab or pembrolizumab for subsequent lines. However, the paradigm has shifted since PD-1 and Programmed Death-Ligand 1 (PD-L1) inhibitors emerged. Landmark studies such as CheckMate 017 and 057 (nivolumab) (23, 24), KEYNOTE-010 (pembrolizumab) (25), and OAK (atezolizumab) (26) established the superiority of immunotherapy over chemotherapy in second-line settings, typically improving median OS by 3–4 months (e.g., 9.2–12.2 months vs. 6.0–9.1 months with chemotherapy).

Tislelizumab is a humanized IgG4 monoclonal antibody that works on PD-1 receptors. It is structurally engineered to minimize binding to Fc gamma receptors (FcγRs) on macrophages, so that it can reduce the antibody-dependent phagocytosis of T-cells. Earlier drugs like atezolizumab, already out there, are humanized antibodies that target PD-L1 instead, stopping it from linking up with PD-1 and B7.1. Literature suggests this modification could improve efficacy by preventing T-cell clearance, a process that might occur with other PD-1 inhibitors (27). While Tislelizumab’s approach is similar to atezolizumab’s in reducing FcγR interactions, its IgG4 base aligns it more closely with nivolumab and pembrolizumab. However, there was no statistically significant difference in OS and PFS outcomes when comparing PD-1 versus PD-L1 agents in extensive-stage SCLC, although subgroup analyses sometimes hinted at modest differences in PFS with first-line treatment favoring PD-1 inhibitors (including tislelizumab) over PD-L1 inhibitors (atezolizumab) in specific populations (28).

Tislelizumab demonstrated a significant PFS improvement by 38% with an HR of 0.62 compared to control treatments. This represents a greater survival benefit compared to pembrolizumab, atezolizumab, and nivolumab, which demonstrated PFS hazard ratios of approximately 0.79, 0.95, and 0.92, respectively, in various trials (23–26). Notably, the PFS benefit observed with tislelizumab is equivalent to that reported in the CHECKMATE 017 trial for squamous NSCLC. Subgroup analysis revealed PFS improvements across lung cancer types, with an HR of 0.61 in NSCLC and 0.65 in SCLC. Among NSCLC subtypes, the benefit was more pronounced in squamous NSCLC, which showed an HR of 0.50 compared to 0.64 in non-squamous NSCLC, surpassing the findings of CHECKMATE 017 PFS.

OS improved by 31% with tislelizumab treatment (HR = 0.69), which is competitive with several major PD-1 inhibitor trials: CHECKMATE 017 (HR = 0.59), CHECKMATE 057 (HR = 0.73), KEYNOTE-010 (HR = 0.61), and OAK (HR = 0.73). DOR was comparable between the tislelizumab and control groups. Notably, the median DOR in the tislelizumab-treated group was approximately 7.69 months, which is substantially shorter than the median DOR reported in other immunotherapy trials, where it typically ranges around 17 months. However, it’s crucial to interpret these results with caution, considering the different follow-up durations, limited sample sizes, and inclusion of various lung cancer types across a small number of studies, such as (19) which included SCLC, while (10) and (18) included mixed squamous and non-squamous NSCLC populations.

The ORR was higher with tislelizumab-based regimens compared to chemotherapy (RR = 1.51), although heterogeneity was substantial (I² = 79.5%). Subgroup analysis by cancer type accounted for a significant portion of this variability. Patients with NSCLC demonstrated a markedly better ORR (RR = 1.72; I² = 59.6%), and further stratification revealed that squamous NSCLC achieved the greatest response (RR = 1.49) with minimal heterogeneity (I² = 0%). These results are consistent with findings from nivolumab trials; however, the present analysis suggests applicability across all lung cancer types, with the strongest effect observed in squamous NSCLC. DCR was initially higher in the tislelizumab group (RR = 1.49), but this outcome demonstrated statistically significant publication bias. Application of the trim-and-fill method to account for potentially missing studies reduced the pooled estimate to a non-significant value, indicating no definitive treatment advantage. Furthermore, the extremely high heterogeneity (I² = 96%) and limited sample size substantially limit confidence in the DCR results.

Current evidence does not include high-quality, double-blind RCTs directly showing that tislelizumab is superior to other PD-1/PD-L1 inhibitors for any lung cancer. However, an indirect comparison evaluated tislelizumab plus chemotherapy versus pembrolizumab plus chemotherapy as first-line treatment for advanced NSCLC, based on ESMO (29)and NCCN guidelines (30). Both combinations significantly improved PFS and ORR compared with chemotherapy alone, with tislelizumab performing similarly to pembrolizumab. These results are in concordance with the findings of network meta-analysis, which showed tislelizumab, pembrolizumab, and nivolumab are therapeutically similar (31), but tislelizumab may provide better PFS across all lung cancer types and a notable reduction in all-cause mortality. Although the indirect evidence appears promising, the lack of direct head-to-head comparisons with Pembrolizumab (32), Atezolizumab (33), and Nivolumab leaves some uncertainty.

Tislelizumab offers a flexible and effective treatment option for both NSCLC and SCLC when combined with chemotherapy. For instance, in the RATIONALE 307 trial, adding tislelizumab to chemotherapy improved PFS for patients with advanced squamous NSCLC, with a median PFS of 7.6 months compared to 5.5 months when only chemotherapy was used (33). Similarly, the RATIONALE 304 trial showed that patients with non-squamous NSCLC had a median PFS of 9.7 months with tislelizumab plus chemotherapy, versus 7.6 months with chemotherapy alone (18). In extensive-stage small cell lung cancer, the RATIONALE-312 trial reported improvements in PFS and OS when tislelizumab was added to chemotherapy (19). Additionally, (21) showed greater PFS benefits than chemotherapy for adenocarcinoma. These findings support our results that tislelizumab can be used effectively as a first-line treatment in lung cancer.

**Safety Considerations:** The overall incidence of side effects with tislelizumab was comparable to control treatments (RR = 1.00, p = 0.75). However, a systematic review suggested that tislelizumab may have a lower risk of severe (grade ≥3) treatment-related adverse events, particularly hematological toxicities, compared to other ICIs (34). Due to limited direct comparisons and reliance on indirect evidence, these findings require further validation. Notably, tislelizumab is significantly associated with elevated AST/ALT levels and significant liver toxicity, a known class effect of PD-1 inhibitors. This immune-mediated liver injury results from excessive T-cell activation and cytokine release (35). Proactive monitoring through regular liver function tests is essential for early detection. Prompt intervention with corticosteroids or immunosuppressants can prevent serious complications (36).

### 4.1 Cost effectiveness

Another interesting finding is related to cost. Some studies have shown that tislelizumab has lower incremental cost-effectiveness ratios compared to atezolizumab and ocrelizumab in China (37), which may make it a more affordable option. Additionally, when compared to docetaxel in NSCLC, tislelizumab showed a favorable cost-effectiveness ratio and quality-adjusted life year gain across the Chinese population (38). Although these findings derive mainly from indirect comparisons and studies focusing on NSCLC or other malignancies rather than direct one-on-one trials with other PD-1/PD-L1 inhibitors, cost considerations could significantly influence treatment selections. Tislelizumab’s recent approval in the United States, currently used for esophageal and hepatocellular carcinomas, and its therapeutic potential for lung cancers may require pricing reforms such as tiered pricing, Medicare negotiations, and improved reimbursement policies to enhance affordability, given the high costs of ICIs. In low- and middle-income countries, ICIs face accessibility challenges due to high upfront costs and limited availability. Tislelizumab’s cost-effectiveness could make it a viable option, but strategies like local production, price controls, and supportive healthcare policies are needed to ensure wider access.

### 4.2 Limitations

This study has certain limitations. While our analysis benefits from including RCTs and different types of lung cancers, mixing different study designs can introduce variations in effect sizes. First, the overall variability was low (I² = 0% for many outcomes), so we performed detailed subgroup analyses to address heterogeneity. However, for DOR, heterogeneity remained high (I² = 76.0%), which could not be explored due to population heterogeneity and limited data (only two studies). Second, most studies were from China, and some had inadequate control of outcome assessments, which may affect the validity. Third, the lack of head-to-head trials with other PD-1/PD-L1 inhibitors also limits the persuasiveness of this study. Other limitations included inconsistent follow-up times, varying chemotherapy regimens, and differences in patient subgroups, which could influence the pooled estimates. Future studies should include larger trials with long-term data on quality of life, late side effects, and more uniform treatment approaches to improve reliability.

### 4.3 Future directions

Larger, rigorously designed RCTs targeting specific lung cancer subtypes and chemotherapy agents are necessary to validate these findings and to identify patient populations most likely to benefit from tislelizumab treatment. Direct comparative trials evaluating tislelizumab against established ICIs such as nivolumab, pembrolizumab, and atezolizumab are essential to establish its relative efficacy. Furthermore, extended longitudinal studies are warranted to assess the durability of clinical responses, the impact on patients’ quality of life, and the emergence of late-onset adverse effects. Future meta-analyses incorporating high-quality observational studies may be valuable for evaluating the external validity of our findings. Additionally, comprehensive cost-effectiveness analyses, alongside detailed biomarker-based stratification including PD-L1 expression and tumor mutational burden, will facilitate precision medicine approaches tailored to individual patients and distinct lung cancer phenotypes.

## 5 Conclusions

This meta-analysis, encompassing 2,148 patients across six studies, demonstrates that Tislelizumab-based regimens offer a statistically significant and clinically relevant benefit over chemotherapy in lung cancer. Tislelizumab was associated with a 38% reduction in PFS and a 31% reduction in risk of death, with minimal statistical heterogeneity. These benefits were consistent across histologies, including ES-SCLC and advanced NSCLC, and aligned with the growing role of PD-1 inhibitors in first-line settings, as reflected in current NCCN and ESMO guidelines. Tislelizumab’s hazard ratios for OS and PFS are comparable to those of established agents such as pembrolizumab and nivolumab. While direct comparisons are limited, its performance in both squamous and non-squamous NSCLC, particularly among patients with PD-L1 expression ≥50%, suggests it is a viable first-line option where accessible, Disease Response and control rates were significantly higher with Tislelizumab-based regimens. Importantly, these clinical benefits were achieved without an increase in overall toxicity. As seen with other immune checkpoint inhibitors, hepatotoxicity, particularly elevated ALT and AST, was more frequent and should be monitored proactively. However, Hematologic adverse events did not differ meaningfully from standard chemotherapy, indicating an overall tolerable safety profile. The evidence base remains modest, and trial heterogeneity, limited blinding, and potential selection biases must be acknowledged. Further head-to-head trials and biomarker-stratified analyses are warranted to define optimal positioning. Nevertheless, for appropriately selected patients, Tislelizumab offers a guideline-concordant, clinically effective, and manageable immunotherapy option across lung cancer subtypes.

## Data Availability

The original contributions presented in the study are included in the article/**Supplementary Material**. Further inquiries can be directed to the corresponding author.
