# Supplementary material for: Comparative safety and efficacy of tislelizumab-based regimens versus chemotherapy in lung cancer: a systematic review and meta-analysis
Source: Front Oncol. 2025 Oct 7;15:1628742. doi: 10.3389/fonc.2025.1628742 (PMC12537389; doi:10.3389/fonc.2025.1628742)

**Supplementary File**

1. **Supplementary Tables**
   1. **Supplementary Table 1.** Search Strategy

| Search Strategy | Tislelizumab OR BGB-A317 OR "tislelizumab" [Supplementary Concept]  Lung cancer OR small cell lung carcinoma OR non-small cell lung carcinoma OR non small cell lung carcinoma OR small cell lung cancer OR non small cell lung cancer OR non-small cell lung cancer  (Antineoplastic Agents OR Chemotherapy OR Chemotherapeutic agent OR Platinum-based chemotherapy OR Platinum chemotherapy OR Chemoradiotherapy)  (Tislelizumab OR BGB-A317) AND (Lung cancer OR small cell lung carcinoma OR non-small cell lung carcinoma OR non small cell lung carcinoma OR small cell lung cancer OR non small cell lung cancer OR non-small cell lung cancer) AND (Antineoplastic Agents OR Chemotherapy OR Chemotherapeutic agent OR Platinum-based chemotherapy OR Platinum chemotherapy OR Chemoradiotherapy) |
| --- | --- |

- 1. **Supplementary Table 2.** Database Search

| **Database** | **(n)** | **Search Strategy** |
| --- | --- | --- |
|  |  |  |
| Pubmed | 47 | Tislelizumab[tw] OR BGB-A317[tw] OR "tislelizumab" [Supplementary Concept]  "Antineoplastic Agents"[Mesh] OR Chemotherap*[tw] OR “Chemotherapeutic agent*”[tw] OR “Platinum-based chemotherapy*”[tw] OR “Platinum chemotherapy*”[tw] OR chemoradiotherap*[tw]  “Lung cancer*”[tw] OR “small cell lung carcinoma”[tw] OR “non-small cell lung carcinoma”[tw] OR “non small cell lung carcinoma”[tw] OR “small cell lung cancer*”[tw] OR “non small cell lung cancer*”[tw] OR “non-small cell lung cancer*”[tw] "Lung Neoplasms"[Mesh] OR "Carcinoma, Non-Small-Cell Lung"[Mesh] OR "Carcinoma, Small Cell"[Mesh]  ((Tislelizumab[tw] OR BGB-A317[tw] OR "tislelizumab" [Supplementary Concept]) AND ("Lung cancer*"[tw] OR "small cell lung carcinoma"[tw] OR "non-small cell lung carcinoma"[tw] OR "non small cell lung carcinoma"[tw] OR "small cell lung cancer*"[tw] OR "non small cell lung cancer*"[tw] OR "non-small cell lung cancer*"[tw] "Lung Neoplasms"[Mesh] OR "Carcinoma, Non-Small-Cell Lung"[Mesh] OR "Carcinoma, Small Cell"[Mesh])) AND ("Antineoplastic Agents"[Mesh] OR Chemotherap*[tw] OR "Chemotherapeutic agent*"[tw] OR "Platinum-based chemotherapy*"[tw] OR "Platinum chemotherapy*"[tw] OR chemoradiotherap*[tw]) |
| Science Direct | 196 | (Tislelizumab OR BGB-A317) AND (Lung cancer OR small cell lung cancer OR non-small cell lung cancer) AND (Antineoplastic Agents OR Chemotherapy OR Platinum chemotherapy OR Chemoradiotherapy) |
| Clinical Trials.gov | 5 | (Tislelizumab OR BGB-A317) AND (Lung cancer OR small cell lung carcinoma OR non-small cell lung carcinoma OR non small cell lung carcinoma OR small cell lung cancer OR non small cell lung cancer OR non-small cell lung cancer) AND (Antineoplastic Agents OR Chemotherapy OR Chemotherapeutic agent OR Platinum-based chemotherapy OR Platinum chemotherapy OR Chemoradiotherapy) |
| Scopus | 134 | (Tislelizumab OR BGB-A317) AND (Lung cancer OR small cell lung carcinoma OR non-small cell lung carcinoma OR non small cell lung carcinoma OR small cell lung cancer OR non small cell lung cancer OR non-small cell lung cancer) AND (Antineoplastic Agents OR Chemotherapy OR Chemotherapeutic agent OR Platinum-based chemotherapy OR Platinum chemotherapy OR Chemoradiotherapy) |
| Embase | 193 | (Tislelizumab OR BGB-A317) AND (Lung cancer OR small cell lung carcinoma OR non-small cell lung carcinoma OR non small cell lung carcinoma OR small cell lung cancer OR non small cell lung cancer OR non-small cell lung cancer) AND (Antineoplastic Agents OR Chemotherapy OR Chemotherapeutic agent OR Platinum-based chemotherapy OR Platinum chemotherapy OR Chemoradiotherapy) |

- 1. **Supplementary Table 3.** Cochrane Search

| **ID** | **(n)** | **Search** |
| --- | --- | --- |
|  |  |  |
| 1 | 339 | (Tislelizumab):ti,ab,kw (Word variations have been searched) |
| 2 | 47 | (BGB-A317):ti,ab,kw (Word variations have been searched) |
| 3 | 352 | #1 OR #2 |
| 4 | 12445 | MeSH descriptor: [Lung Neoplasms] explode all trees |
| 5 | 667 | MeSH descriptor: [Small Cell Lung Carcinoma] explode all trees |
| 6 | 6844 | MeSH descriptor: [Carcinoma, Non-Small-Cell Lung] explode all trees |
| 7 | 34180 | (Lung cancer OR small cell lung carcinoma OR non-small cell lung carcinoma):ti,ab,kw (Word variations have been searched) |
| 8 | 35223 | #4 OR #5 OR #6 OR #7 |
| 9 | 20070 | MeSH descriptor: [Antineoplastic Agents] explode all trees |
| 10 | 2208 | MeSH descriptor: [Chemoradiotherapy] explode all trees |
| 11 | 110840 | (Antineoplastic Agents OR Chemotherapy OR Platinum-based chemotherapy OR Chemoradiotherapy):ti,ab,kw (Word variations have been searched) |
| 12 | 113627 | #9 OR #10 OR #11 |
| 13 | 106 | #3 AND #8 AND #12 |

1. **Supplementary Figures**

**2.1. Supplementary Figure 1.**

**
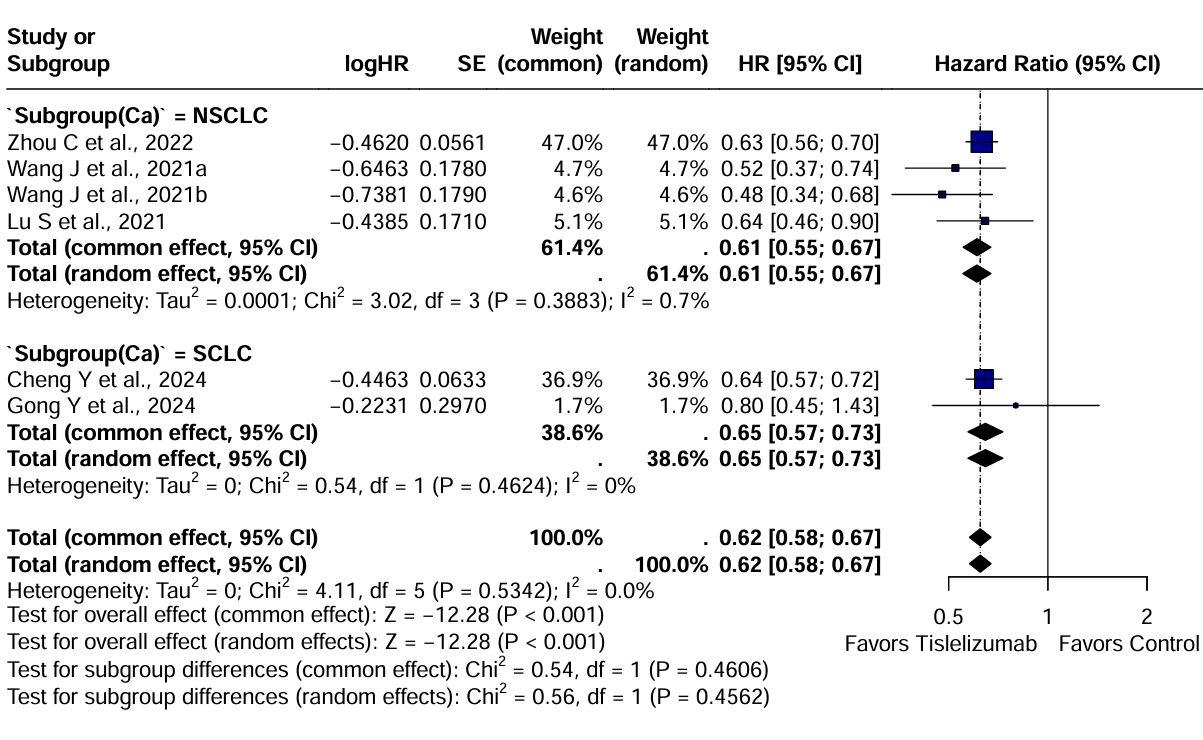

2.2. Supplementary Figure 2.**

**
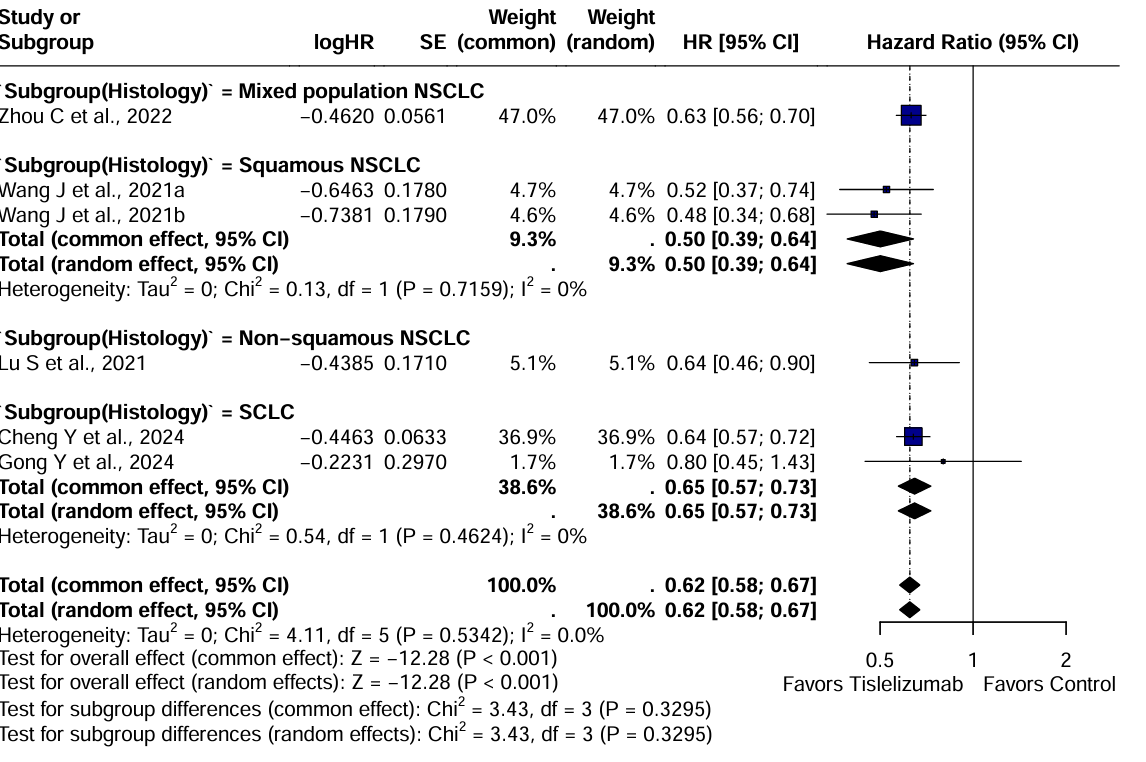
**

**2.3. Supplementary Figure 3
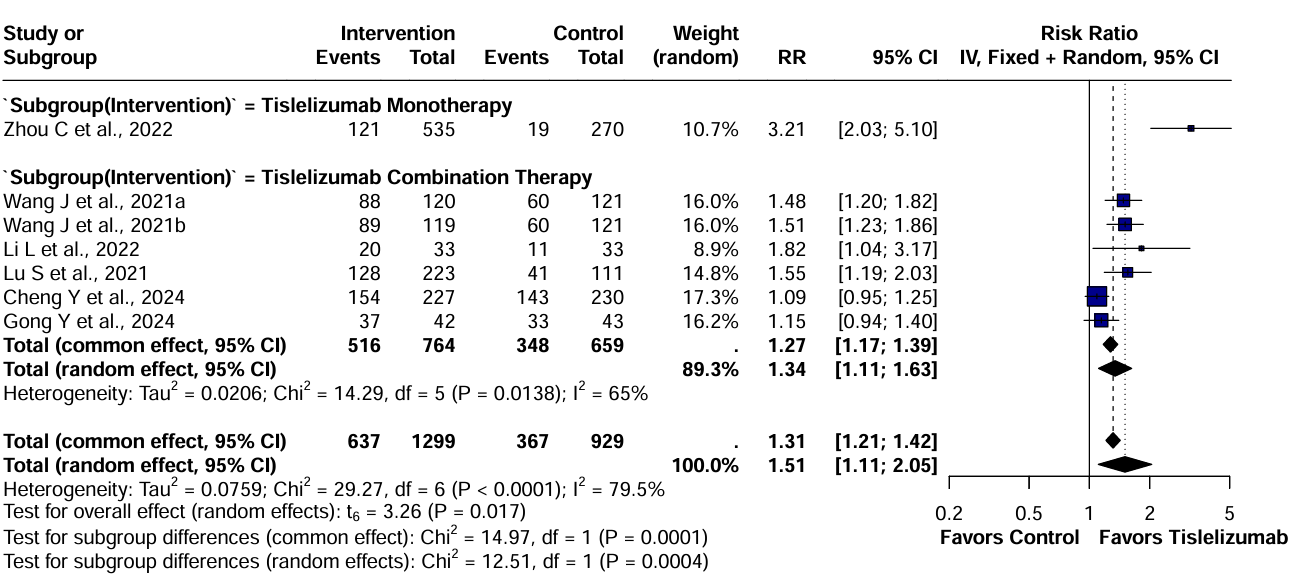
**

**2.4. Supplementary Figure 4.**

**
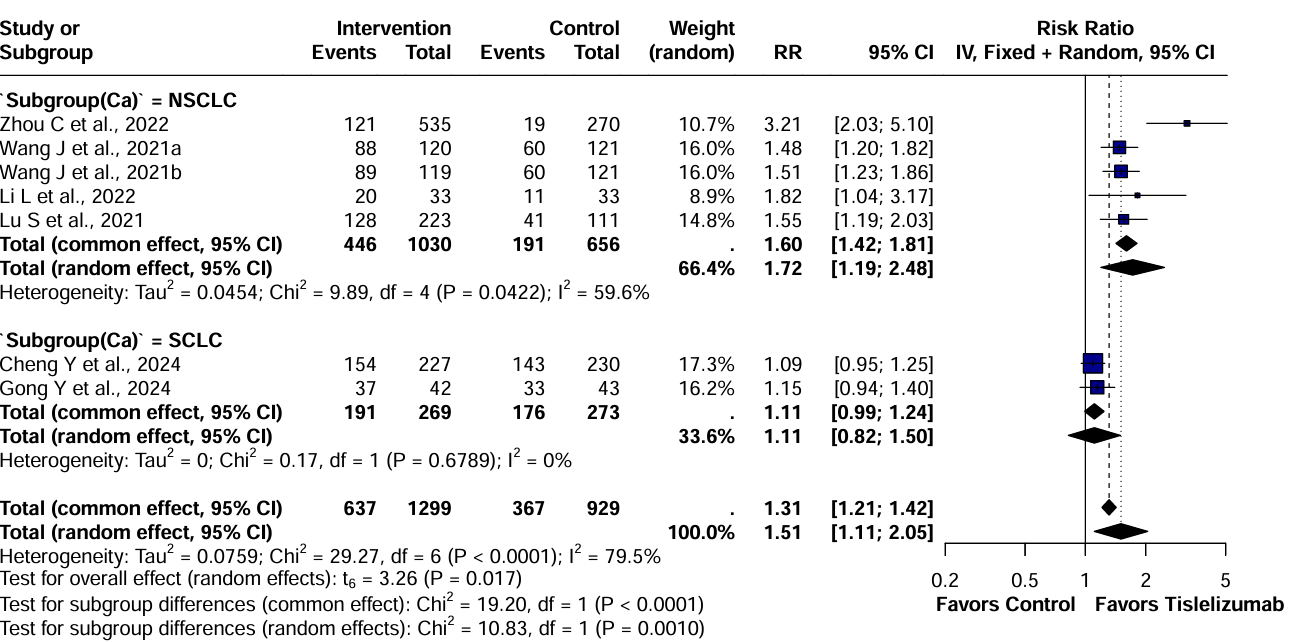
**

**2.5. Supplementary Figure 5.**

**
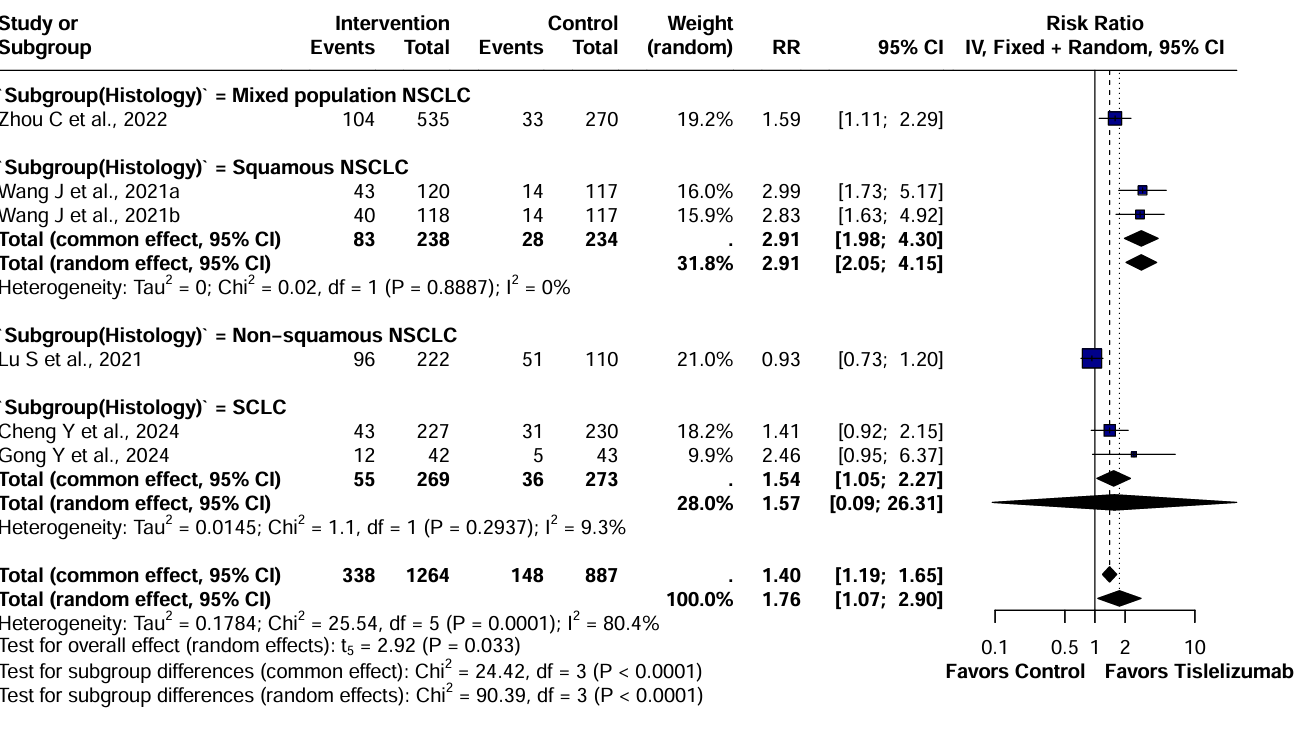
**

**2.6. Supplementary Figure 6. Quality Assessment of Studies**


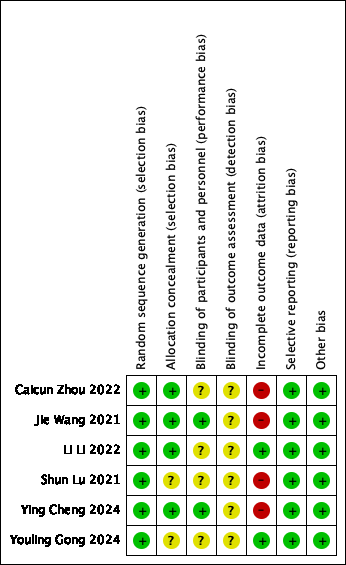


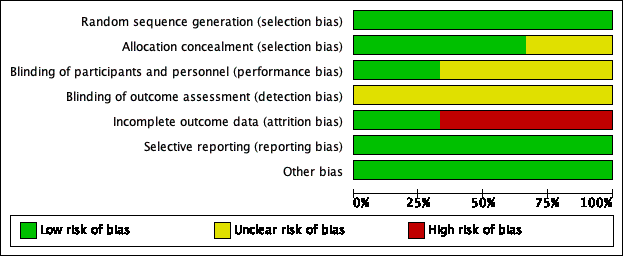


**2.7 Supplementary Figure 7.** Forest Plot for AST alongwith sensitivity analyses across included studies


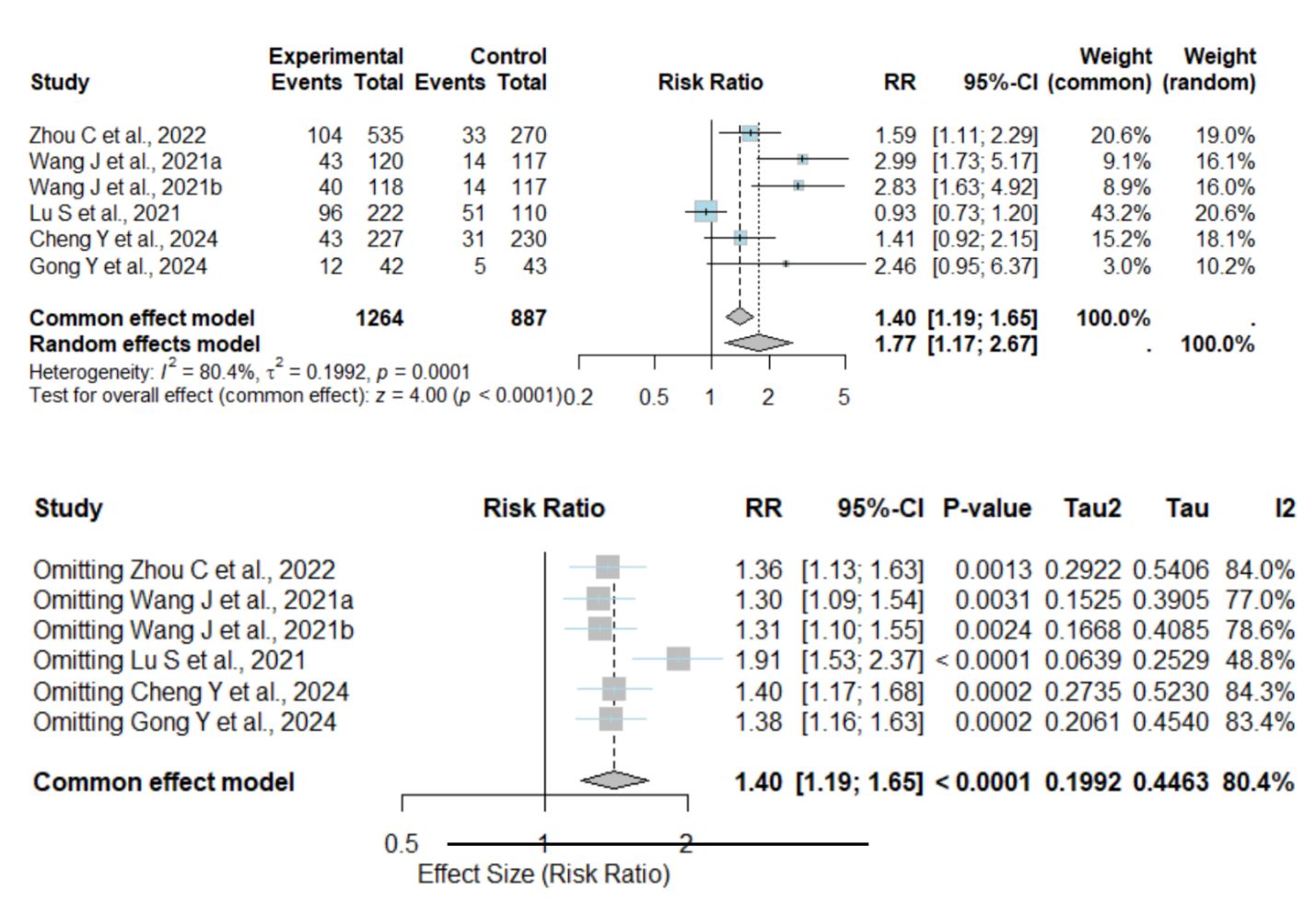

Supplement: Supplementary file 1 [file DataSheet1.docx]
